# Orchid Reintroduction Based on Seed Germination-Promoting Mycorrhizal Fungi Derived From Protocorms or Seedlings

**DOI:** 10.3389/fpls.2021.701152

**Published:** 2021-06-30

**Authors:** Da-Ke Zhao, Marc-André Selosse, Limin Wu, Yan Luo, Shi-Cheng Shao, Yong-Ling Ruan

**Affiliations:** ^1^Biocontrol Engineering Research Center of Plant Disease and Pest, Biocontrol Engineering Research Center of Crop Disease and Pest, School of Ecology and Environmental Science, Yunnan University, Kunming, China; ^2^Département Systématique et Evolution, UMR 7205 ISYEB, Muséum National d'Histoire Naturelle, Paris, France; ^3^Faculty of Biology, University of Gdansk, Gdansk, Poland; ^4^Agriculture and Food, Commonwealth Scientific and Industrial Research Organisation, Canberra, ACT, Australia; ^5^Gardening and Horticulture Department, Xishuangbanna Tropical Botanical Garden, Chinese Academy of Sciences, Mengla, China; ^6^Australia-China Research Centre for Crop Improvement, School of Environmental and Life Sciences, The University of Newcastle, Callaghan, NSW, Australia

**Keywords:** symbiosis, seed germination, reintroduction, orchid mycorrhizal fungi, orchid conservation

## Abstract

Orchids are among the most endangered in the plant kingdom. Lack of endosperm in their seeds renders orchids to depend on nutrients provided by orchid mycorrhizal fungi (OMF) for seed germination and seedling formation in the wild. OMF that parasitize in germination seeds is an essential element for orchid seedling formation, which can also help orchid reintroduction. Considering the limitations of the previous orchid reintroduction technology based on seed germination-promoting OMF (sgOMF) sourced from orchid roots, an innovative approach is proposed here in which orchid seeds are directly co-sown with sgOMF carrying ecological specificity from protocorms/seedlings. Based on this principle, an integrative and practical procedure concerning related ecological factors is further raised for re-constructing long-term and self-sustained orchid populations. We believe that this new approach will benefit the reintroduction of endangered orchids in nature.

## Orchid Reintroduction and Conservation: A Global Urgency

Orchidaceae is the second largest family of flowering plants after Asteraceae (Chase et al., 2015; Givnish et al., 2015; Willis, 2017), with a total of 29,199 species identified (Govaerts et al., 2017). They are tremendously valuable for biodiversity, conservation, and the production of a wide range of medicinal compounds, healthy food, and ornamental plants (Willis, 2017; Hinsley et al., 2018). Moreover, they are popular flagships for habitat conservation. However, orchids are currently among the most threatened flowering plants, with many species on the verge of extinction in the wild due to over-collection, loss of habitats, or climate change (Liu et al., 2015; Gale et al., 2018; Hinsley et al., 2018; Wang et al., 2019). Furthermore, most orchids require specialized habitats and are usually in small populations with a high dependence on pollinators, symbiotic germination fungi, and host trees for epiphytic species, further making them particularly vulnerable to extinction (Roberts and Dixon, 2008; Selosse, 2014; Fay et al., 2015; Rasmussen et al., 2015; Keppel et al., 2016; Fay, 2018; Gale et al., 2018).

Retrospectively, few orchid species have been domesticated and cultivated on a large scale, except for these with high values in ornamental horticulture (e.g., in the genera *Cymbidium, Phalaenopsis*, and *Cattleya*), medicine (several *Dendrobium* and *Gastrodia* spp.) or food industries (e.g., *Vanilla fragrans*). Consequently, a large portion of orchids still grows in their natural habitats. All orchids have been included in their entirety in Appendices I and II of the Convention on International Trade in Endangered Species of Wild Fauna and Flora (CITES) in 2017 to ban illegal trade (Gale et al., 2018; Hinsley et al., 2018). Based upon the assessments of 1770 orchid species worldwide in 2021 by Global International Union for Conservation of Nature (IUCN) for Red Lists of Threatened Species, nearly a half (46.5%) of orchid species are under one of the three threat categories, namely, vulnerable, endangered, and critically endangered (IUCN, 2021). Clearly, the majority of orchids are facing extinction threats, which demands urgent attention and targeted conservation actions such as reintroduction (Roberts and Dixon, 2008; Rasmussen et al., 2015). Reintroduction means the controlled placement of plant individuals of an endangered species into its natural habitat or managed ecological area to re-establish populations in the wild.

## Reintroduction as a Priority for Efficient Orchid Conservation

*Ex situ* and *in situ* conservation, together with reintroduction, are the main methods for the conservation of threatened plant species (Oldfield, 2009). For plants deprived of their original natural habitats, *ex situ* conservation in botanic gardens may be the only way for their survival in short to medium terms (Ren et al., 2014). Of the 350,699 categorized plant species, 105,634 or 30% are held in the living collections of the global botanic garden network, indicating a significant effort in *ex situ* conservation (Mounce et al., 2017). Botanic gardens are, however, are concentrated on temperate regions and a majority of the collected species is kept in the northern hemisphere (Mounce et al., 2017). It implies that a large number of orchids is beyond *ex situ* conservation, mostly growing in tropical and subtropical areas with high genetic diversity (Givnish et al., 2015).

In contrast to *ex situ* conservation, *in situ* conservation is considered to be more effective for sustainable biodiversity conservation. The procedure is, however, complex and multifaceted. It involves both the maintenance and management of the protected areas and actions required at the species and population levels. So far, species-level measures have only been undertaken for a very small percentage of threatened plants by a few countries, and with limited success (Heywood, 2015). Indeed, it is difficult to carry out efficient *in situ* conservation for most of the endangered orchids due to the vast number of species involved and their broad geographic distributions.

Reintroduction of individual plants to their natural habitats, as an essential and effective measure to protect endangered species, has become increasingly important for species conservation worldwide, especially for plants facing extinction such as many orchid species (Oldfield, 2009; Godefroid et al., 2011). Plants from *ex situ* conservation can be linked to *in situ* conservation via reintroduction. In other words, reintroduction could bridge the gap between conservation theories and practices (Gale et al., 2018). As a consequence, reintroduction programs represent a better choice for orchid protection (Zeng et al., 2012; Wu et al., 2014).

## Seed Germination Promoting Orchid Mycorrhizal Fungi (sgOMF) Play Key Roles in the Orchid Reintroduction

Orchid conservationists have long been trying to develop ways to reintroduce endangered orchids (Swarts and Dixon, 2009; Johnson, 2011). The current strategies of orchid reintroduction primarily focus on restoration-friendly cultivation, translocation of individual plants, or transplantation of *in vitro* cultured seedlings (Zeng et al., 2012; Wu et al., 2014). However, these approaches have often been proven to be insufficient and ineffective for *in situ* protection of some critically endangered orchid species, because of the poor survival rate of the seedlings produced *in vitro* or the low genetic diversity of the reintroduced plants (Liu et al., 2010; Shao et al., 2017; Hinsley et al., 2018; Sathiyadash et al., 2020).

Due to the lack of endosperm (Yeung, 2017; Zhang et al., 2017; Yeh et al., 2019), the germination of nearly all orchid seeds relies on the specific fungal partner in the wild (Bruns and Read, 2000; Bidartondo and Read, 2008; Merckx et al., 2009; Swarts and Dixon, 2009; Dearnaley et al., 2012; McCormick et al., 2018; Shao et al., 2019). To this end, the seed germination-promoting fungi, predominantly residing in the protocorm of the germinating seeds are known to play a key role in realizing *in situ* orchid seed germination and seedling formation (Selosse et al., 2017). Seed germination-promoting fungi generally belong to orchid mycorrhizal fungi (OMF) characterized by the formation of pelotons, inside the cells of orchid roots or germinating seeds (Jacquemyn et al., 2017). Therefore, we focus on sgOMF for orchid reintroduction.

**Figure 1:**
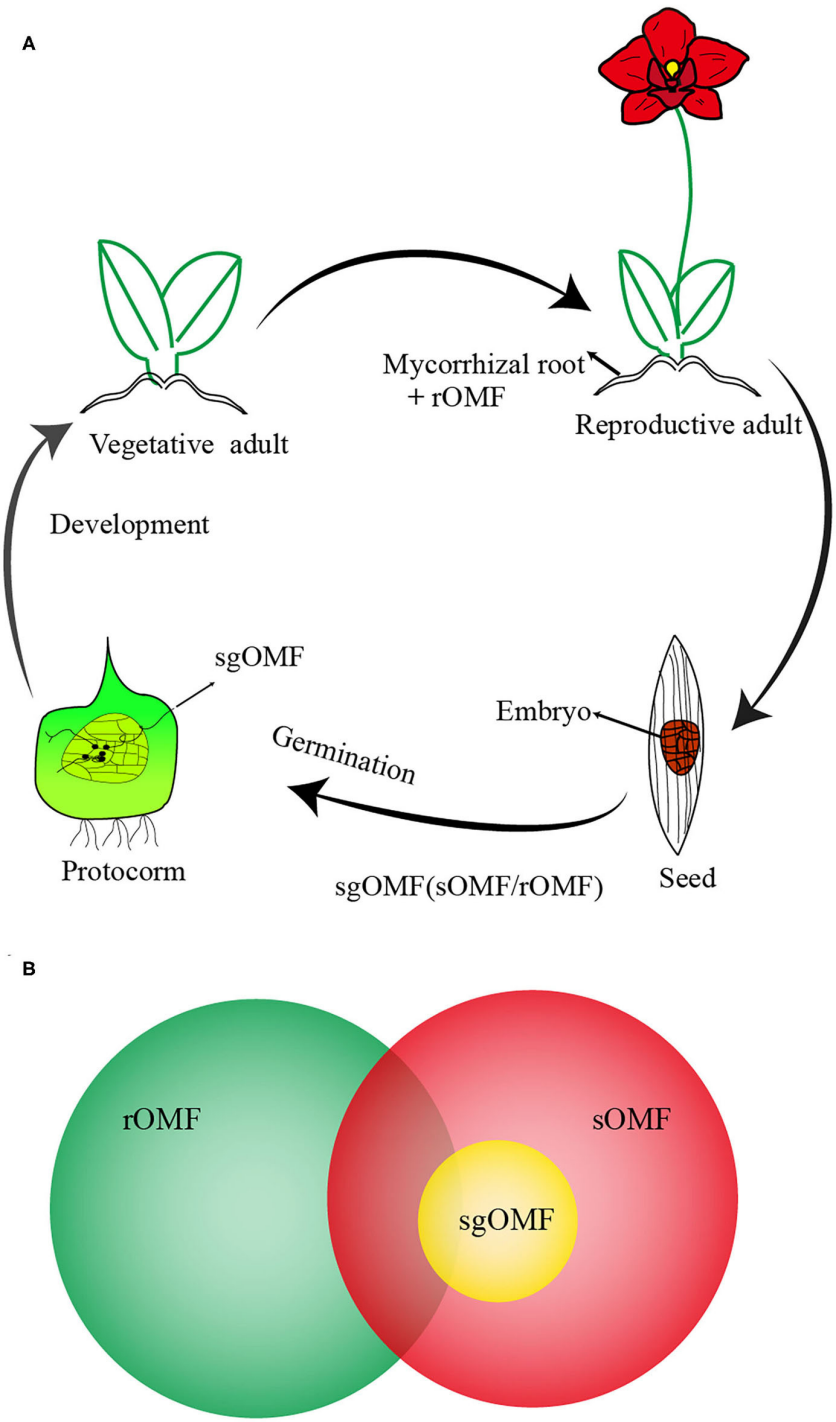
A schematic presentation on the role of rOMF, sOMF, and sgOMF in orchid life cycle and their relationship. **(A)** A flow chart on orchid life cycle from seed germination to adult plants with positions of rOMF and sOMF in the life cycle indicated. Due to the lack of endosperm, orchid seed germination requires the establishment of symbiosis with seed germination-promoting OMF (sgOMF), mostly found in the protocorm of the germinating seeds (sOMF). Vegetative adults can develop into the reproductive stage and undergo mycorrhization with root-originated OMF (rOMF). **(B)** A schematic diagram on the relationship among rOMF, sOMF, and sgOMF. Both rOMF and sOMF originate from saprophytic or ectomycorrhizal fungi. Here, sgOMF is the subset of sOMF capable of promoting orchid seed germination *in situ*. rOMF strains are often different but could be similar to sOMF (see text for more details).

In broad term, OMF could colonize orchid roots (root-originated OMF, rOMF) or seeds (seed-originated OMF, sOMF) at distinctive development stages as illustrated in Figure 1A (Peterson et al., 2004; Smith and Read, 2008; Yeh et al., 2019; Favre Godal et al., 2020; Sathiyadash et al., 2020). Theoretically, sgOMF could be potentially identified from sOMF or rOMF (Figure 1). There may be more than one OMF species colonized at a given germinating stage (Bidartondo and Read, 2008; Stöckel et al., 2014; Shao et al., 2017, 2019; Meng et al., 2019a). These sgOMF are saprotrophic and/or endophytic in non-orchid plants (Selosse and Martos, 2014; Weiβ et al., 2016), or concurrently ectomycorrhizal (typical for terrestrial or mycoheterotrophic orchids).

At the genus level, sgOMF generally belong to the polyphyletic “rhizoctonia” aggregate that includes Tulasnellaceae, Ceratobasidiaceae, and Serendipitaceae (Dearnaley et al., 2012; Rasmussen and Rasmussen, 2014; Rasmussen et al., 2015). Some other genera are reported to be involved in symbiotic germination as well, including *Mycena* (Xu and Guo, 1989), *Helicogloea* (Kottke et al., 2010), *Thanatephorus* (Sebastian et al., 2014), and *Fusarium* (Jiang et al., 2019). Beyond saprobic fungi, some mycoheterotrophic orchid species display symbiosis with *Tomentella, Russula, Tuber*, or other fungi (Selosse et al., 2004; Julou et al., 2005; Abadie et al., 2006; Bidartondo and Read, 2008; Fochi et al., 2017; Shao et al., 2017).

It was believed that sgOMF generally come from rOMF (e.g., Rasmussen, 1995). Yet, although this may apply to many terrestrial orchids from temperate regions, the general validity of this hypothesis deserves a close examination. At the species level, sgOMF may or may not be identical to those from the corresponding adult roots (McCormick and Jacquemyn, 2014; Meng et al., 2019c). Even for the same fungal species, the isolates from adult roots often fail to promote seed germination *in situ*, while that fungi from protocorms can (Zelmer and Currah, 1997). The underlying mechanism for this disparity remains to be elucidated (Douhan et al., 2011; Johnson et al., 2012).

The mycorrhizal specificity associated with an orchid *in situ* seed symbiotic germination is often so restrictive that seeds from many orchids do not germinate or develop without their compatible fungal symbiont (Rasmussen, 1995; Bruns and Read, 2000; Merckx et al., 2009; Tesitelova et al., 2012; Davis et al., 2015; Fay, 2018). Even if seed germination is triggered by close relatives of the target fungus, the orchid seedling may not survive beyond the early developmental stages, which probably explains why some protocorms succeed in germination but fail in further seedling growth. The exact cause of this extreme level of fungal specificity under *in situ* symbiotic germination is yet to be examined. Nevertheless, coevolution might play a role in this phenomenon: once an appropriate fungus had been recruited. The orchid may have fine-tuned its physiology to adapt to this fungus, thereby making the plants incapable of host-jumping for distantly related fungi (Bidartondo and Bruns, 2002), especially under *in situ* conditions (Masuhara and Katsuya, 1994; Perkins et al., 1995).

Some sOMF and rOMF can promote orchid seed germination under controlled laboratory conditions, i.e., *in vitro*, thereby exhibiting potential specificity (Smith and Read, 2008; Rasmussen et al., 2015; Jacquemyn et al., 2017; Shao et al., 2019; Sathiyadash et al., 2020). If those isolates exhibiting potential specificity can successfully stimulate orchid seed symbiotic germination and survival under *in situ* conditions, they are considered to have ecological specificity. Mycorrhizal roots are historically the major source for identifying fungi that could be used for orchid symbiotic seed germination, since it is much easier to access rOMF than sOMF from protocorms or germinating seeds. The latter are difficult to be detected under natural conditions (Perkins et al., 1995; Zelmer and Currah, 1997; Steinfort et al., 2010; Herrera et al., 2017; Jiang et al., 2019). Potential specificity may also be possessed by some endophytic or even soil fungi (Jusaitis and Sorensen, 1993; Vujanovic and Vujanovic, 2007; Jiang et al., 2019). However, the effects of rOMF isolates with potential specificity have rarely been evaluated in natural environments or *in situ*. Among those tested, few of them showing ecological specificity in the wild where the natural factors are uncontrollable and much more complex and variable than those *in vitro* (Meng et al., 2019c).

While sgOMF may exist among the rOMF (Masuhara and Katsuya, 1994; Bidartondo and Read, 2008; Stöckel et al., 2014), the isolates from adult roots do not always display the ability to promote seed germination. For example, among the *Ceratobasidium cornigerum* recovered from the field-grown adult roots and the protocorms of *Spiranthes lacera* at the same site, only the strain from the protocorm, promoted seed germination and seedling development (Zelmer and Currah, 1997). Furthermore, even if the isolate from adult roots promotes *in vitro* germination and seedling formation, it could fail to work in the field (Batty et al., 2006).

Due to the lack of or lower chance of finding sgOMF from roots, the effective way is to isolate sgOMF from the protocorms or germinating seeds either occurred naturally or induced using *in* or *ex situ* baiting. This method allows simultaneous baiting for mycorrhizal fungi that promote orchid seed germination *in situ* or *ex situ* from soil or bark (Shao et al., 2017). Symbiotic fungal diversity is sometimes lower in protocorms and seedlings than that in the adult roots (Bidartondo and Read, 2008; Zi et al., 2014). The sOMF usually consist of few fungal strains per germinating seed or seedling and in most cases display a high degree of species-specificity (Masuhara and Katsuya, 1994; Rasmussen and Rasmussen, 2014). However, one should not overlook the possibility that multiple sgOMF species may colonize protocorms or seedlings (Perkins et al., 1995; Bidartondo and Read, 2008; Tesitelova et al., 2012; Stöckel et al., 2014; Meng et al., 2019a; Shao et al., 2019, 2020). Therefore, candidate sgOMF should be firstly evaluated for their potential specificity for seed germination *in vitro* (Masuhara and Katsuya, 1994; Jacquemyn et al., 2017).

## Limitations of the Current Orchid Reintroduction Relying on rOMF

The concept of using rOMF to promote orchid seed germination was proposed by Bernard in the early 1900's (Selosse et al., 2017) and elaborated later by others including Warcup and Clements in the 1970's and 1980's (Clements et al., 1986). Rasmussen (1995) and Zettler (1997) then suggested the application of rOMF in orchid reintroduction about 20 years ago. There are several preliminary reintroduction reports with rOMF for some green terrestrial orchids including *Spiranthes magnicamporum* (Anderson, 1991), *Spiranthes brevilabris* (Stewart et al., 2003), *Dactylorhiza hatagirea* (Aggarwal and Zettler, 2010), and *Dactylorhiza praetermissa* (Mckendrick, 1995), two epiphytic orchids *Epidendrum nocturnum* (Zettler et al., 2007) and *Vanda coerulea* (Aggarwal et al., 2012) (Table 1). Despite the progress and the role that rOMF play in nutrient uptake from soil (Dearnaley et al., 2012) and vegetative dormancy (McCormick et al., 2018), there are several major limitations of the rOMF-based reintroduction.

**Table 1:** Key orchid reintroduction cases based upon OMF.

**Taxa**	**OMF**	**OMF origin**	**Specificity**	**Materials for reintroduction**	**Supervision period**	**References**
*Spiranthes magnicamporum*	*Epulorhiza repens*	Roots	Potential specificity	Seedling	Flowering after planted on calcarious sand 15 months later	Anderson, 1991
*Dactylorhizapraetermissa*	*Unidentified fungus*	Roots	Potential specificity	Seedlings	Flowering after 2 or 3 years	Mckendrick, 1995
*Spiranthes brevilabris*	*Epulorhiza repens*	Roots	Potential specificity	Seedlings	Flowering after planted 6 months later	Stewart et al., 2003
*Epidendrumnocturnum*	*Epulorhiza repens*	Roots of *Spiranthes brevilabris*	Potential specificity	Seedlings	Not mentioned	Zettler et al., 2007
*Dactylorhiza hatagirea*	*Ceratobasidium* sp.	Roots	Potential specificity	Seedlings	Individuals without flowering after 2 years later	Aggarwal and Zettler, 2010
*Vanda coerulea*	*Thanatephorus cucumeris*	Roots	Ecological specificity	Seedlings	Individuals after 1 year	Aggarwal et al., 2012
*Dendrobium devonianum*	*Tulasnella* sp.	Protocorms	Ecological specificity	Co-sowing seeds and *tulasnella* sp.	Seedlings after 3 month	Shao et al., 2017
*Dendrobium aphyllum*	*Tulasnella calospora*	Protocorms	Ecological specificity	Co-sowing seeds and *tulasnella calospora*	Seedlings after 3 month	Shao et al., 2018
*Dendrobium nobile*	*Tulasnella* sp.	Protocorms	Ecological specificity	Co-sowing seeds and *tulasnella* sp.	Seedlings after 3 month	Shao et al., 2018

Firstly, based on available information, this method hardly led to the re-establishment of a self-sustainable population for critically endangered orchids (Scade et al., 2006). The success of long-term reintroduction can only be achieved if self-sustained populations are established that do not require further human intervention (Scade et al., 2006; Reiter et al., 2016). Orchid mycorrhizal associations are essential for rebuilding self-sustainable wild populations (Scade et al., 2006; Liu et al., 2010; Reiter et al., 2016). Noteworthily, it is the sgOMF from sOMF, but not rOMF, often promote *in situ* seed germination and the subsequent survival and adaption to the natural habitats (Batty et al., 2006; Shao et al., 2017). In other words, sgOMF can be selected among fungi colonizing protocorms (Shao et al., 2017, 2018), whereas the rOMF in many cases lack the ecological specificity required and fail to help the rebuilding of self-sustained orchid populations.

Some rOMF strains promote seed germination and plantlet formation under laboratory conditions, thereby exhibiting potential specificity (Steinfort et al., 2010; Herrera et al., 2017; Jiang et al., 2019). Some of those individuals even reach the reproductive stage after being transplanted from *in vitro* environment to the field (Mckendrick, 1995; Stewart et al., 2003). However, and although this was rarely checked, rOMF may not possess suitable inoculum of sgOMF from protocorms and may lack ecological specificity.

Secondly, there is a risk of a failure in identifying any sgOMF from adult orchid roots. For example, all rOMF isolates (*Tulasnella* spp.) failed to sustain seedling growth *in vitro* both in the epiphytic *Dendrobium exile* and terrestrial *Arundina graminifolia* (Meng et al., 2019b,c). Overall, the limitations and challenges outlined above make rOMF approach impractical for *in situ* orchid reintroduction (Reiter et al., 2016).

It is clear that although the application of rOMF contributed to reintroduction in the past and may continue to do so in the future, it has several inherent limitations that prompt efforts to develop new approaches. Moreover, the specificities of epiphytic orchids, both in terms of ecology and fungal partners (Martos et al., 2012), call for re-thinking the protocols inherited from the study of North-American and European terrestrial orchids.

## Targeting sgOMF From Protocorms and Germinating Seed for Successful *in situ* Orchid Reintroduction

Under field conditions, sOMF often promote symbiotic seed germination *in situ*, resulting in the development of plantlets (Masuhara and Katsuya, 1994; Perkins et al., 1995; Smith and Read, 2008; Sathiyadash et al., 2020) (Figure 1), thereby contributing to the rebuilding the orchid populations in the wild. Due to the ecological specificity displayed by sOMF for the symbiotic germination of the target orchid (Perkins et al., 1995; Shao et al., 2017, 2018; Meng et al., 2019c), orchid reintroduction based on sOMF is a natural way compared to that of rOMF. By co-sowing orchid seeds with germination-enhancing sgOMF carrying ecological specificity (see below), the resultant plantlets could serve as not only orchid individuals but also inoculum in the surroundings of the target orchid as a germination promoting- mycobiont for continuous orchid germination (Batty et al., 2006; Reiter et al., 2016). It thereby helps to re-establish self-sustainable populations for critically endangered orchids, particularly for those orchids whose *ex situ* conservation should be conducted in the wild.

Protocorms and seedlings simultaneously associate with multiple OMF in the wild (Shao et al., 2019, 2020). However, co-cultures with two or three sgOMF did not increase protocorm formation and seedling establishment. Rather it often resulted in lower germination percentages compared with that of monocultures for *D. nobile* (Shao et al., 2020). While it seems easy to just perform direct sowing of orchid seeds into their natural habitats for reintroduction, this practice typically leads to sporadic and unstable seedling emergence, owing to the low probability of encountering the target sgOMF in the environment (Jacquemyn et al., 2007; Jersakova and Malinova, 2007; Zi et al., 2014; Shao et al., 2017; Yang et al., 2017).

The concept to use sgOMF carrying ecological specificity for orchid rehabilitation was raised 25 years ago (Perkins et al., 1995). Two decades later, the application of sgOMF from sOMF in orchid restoration finally came into reality for the epiphytic orchid *Dendrobium devonianum* (Shao et al., 2017). Using packaged seed mixed with symbiotic fungus *Tulasnella* sp. isolated from protocorms, an efficient reintroduction was conducted, which resulted in the construction of a natural population of this endangered orchid (Shao et al., 2017). With improved methods, the other two threatened orchid species *Dendrobium aphyllum* and *D. nobile* have also been successfully reintroduced to the wild, as well as the restoration of another field population of *D. devonianum* (Shao et al., 2018).

Since epiphytes account for ~70% of all the orchid species (Peterson et al., 2004; Zotz, 2013) and previous reintroduction activities largely focused on terrestrial orchids (Anderson, 1991; Mckendrick, 1995; Stewart et al., 2003; Aggarwal and Zettler, 2010), the co-sowing practice to construct populations for these epiphytic species (Shao et al., 2017, 2018) represents a promising direction and tool in orchid population establishment. Based on the success in the reintroduction of the three epiphytic orchids (Shao et al., 2017, 2018) as discussed above, it is now the time for the application of sgOMF carrying ecological specificity to be tested on other genera. We presume that it will be an effective way to achieve orchid reintroduction *in situ*.

In contrast to the use of rOMF to produce seedlings *ex situ*, the reintroduction of the three *Dendrobium* spp. was based on a novel approach where orchid seeds were directly germinated *in situ* with the resultant seedlings fully developed into fertile adult plants (Shao et al., 2017, 2018) (Table 1). As nearly all the orchids that naturally germinate are accompanied with sgOMF with ecological specificity, the sOMF-based approach could be broadly applicable for the efficient reintroduction and even natural cultivation of endangered orchidaceae species. One should bear in mind that regardless the rOMF- or sOMF- based studies, there have been no reports on long term analyses over 5 years to check the status of reintroduction if the plantlets emerged from seeds. Thus, monitoring the seedling recruitment over two generations is needed to further verify the result of orchid reintroduction with sOMF (Table 1), especially when reintroducing to sites where the orchids disappeared long time ago. We described the key steps involved in this procedure exploiting sgOMF isolated from protocorms or germinating seeds.

## Isolation of sgOMF From Protocorms or Germinating Seeds With Potential Specificity

**Figure 2:**
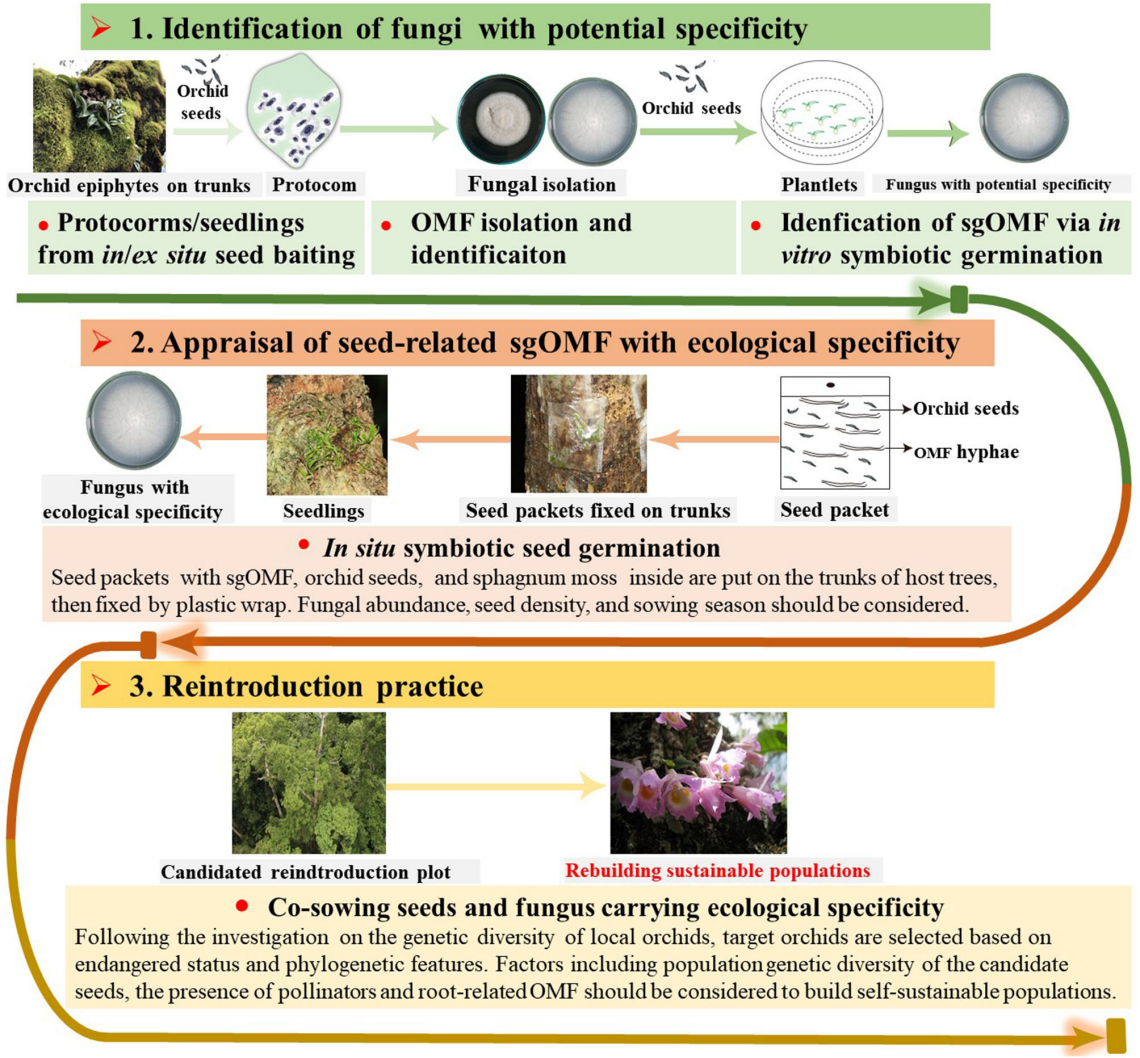
A procedure for *in situ* orchid reintroduction through direct co-sowing with sgOMF. The protocol consists of three key steps: (i) Identification of fungal strains with potential specificity isolated from protocorms or germinating seeds using symbiotic germination *in vitro*; (ii) Screening for sgOMF carrying ecological specificity through seed-fungus co-sowing *in situ*, and (iii) Reintroduction. More specifically, *in* or *ex situ* seed baiting is recommended to develop protocorms and germinating seeds slightly beyond the protorm stage, from which sgOMF candidates are isolated. All isolates are screened for their efficiency in promoting seed germination *in vitro* in laboratories, leading to the identification of sgOMF with potential specificity. To further identify sgOMF carrying ecological specificity, paper bags containing sgOMF candidates, orchid seeds, and sphagnum moss are placed on the trunks of host trees under *in situ* conditions. With the successful identification sgOM exhibiting ecological specificity, reintroduction can proceed (see text for more details).

To realize *in situ* orchid reintroduction, it is essential to identify sgOMF carrying ecological specificity for initiating symbiotic seed germination *in situ*. As mentioned before, sgOMF isolated from protocorms or seedlings typically promote seed germination not only *in vitro* but also in the wild, thus showing both potential and ecological specificity (Shao et al., 2017, 2018). The isolates are first verified for potential specificity in laboratories (Zi et al., 2014; Shao et al., 2017, 2019; Meng et al., 2019c) (Figure 2). Yet, sgOMF exhibiting potential specificity does not necessarily carry ecological specificity; as that if they could stimulate *in situ* seed germination remains unclear. Thus, further screening is required.

## Appraisal of Ecological Specificity of sgOMF From Protocorms or Germinating Seed

After having isolated sgOMF with potential specificity, their *in situ* co-culture with orchid seeds are conducted to screen for sgOMF capable of promoting seed germination in natural habitats, i.e., carrying ecological specificity (Figure 2). The procedure is supposed to be easily carried out by blending orchid seeds with mycelium and subsequently transferring the mixture to the wild. In reality, however, it faces great challenges due to the high complexity of orchid seed germination *in situ* (Rasmussen et al., 2015). Hence, innovative and comprehensive measures are needed to design an efficient, reliable, and practical program. Based on our experience in conjunction with considerations of other factors (Shao et al., 2017, 2018), we summarized below the key conditions influencing *in situ* symbiotic germination.

For epiphytic orchids, maintaining moisture is crucial for achieving *in situ* seed germination. Previously, orchid seeds were placed on organic substances such as sphagnum moss or bark for germination (Arditti, 1967), which often results in a disappointing outcome largely because of the dry microenvironment surrounding the seeds. To circumvent this problem, paper packets containing a mixture of orchid seeds and fungal hyphae were attached to tree trunks and then wrapped with biodegradable plastic film to retain moisture for producing seedling *in situ* (Shao et al., 2017). Further supplement of sphagnum moss in paper packets increased the germination rate of *D. devonianum* to 9.4% (Shao et al., 2018), as compared to only 0.9~1.4 % without the inclusion of the moss (Shao et al., 2017). We consider that this 10-fold increment in the germination rate is primarily owing to the much-improved moisture retention during early germination. Moreover, instead of using nylon net packets in primary reintroduction studies, which could physically block late seedling growth (Shao et al., 2017, 2018), the paper packets did not constrain seedling emergence. The seedlings can grow readily by enlarging the holes punctured in the wrap, allowing the plantlets to develop naturally without the need for other management intervention (Shao et al., 2018).

Sowing time is another important factor impacting germination *in situ* as it relates to air temperature and humidity (Shao et al., 2017, 2018). *In situ* seed germination and the subsequent formation of protocorm-like bodies and seedlings correlate with the humidity as well as the temperature of the microhabitats across seasons (Shao et al., 2017, 2018; Yang et al., 2017). Therefore, knowledge on the local timing of natural seed dispersal and their germination of the target orchid is required to determine sowing time.

The density of the fungal patch could also affect orchid seed germination (McCormick et al., 2012, 2018; Favre Godal et al., 2020). The mixture with enough pre-grown fungal hyphae with orchid seeds keeps the dominance of sgOMF, preventing the invasion of competitors (Shao et al., 2017). In practice, fungal mycelium mixed with agar was blended into powder and used at the ratio of 1.0 mg powder to 50 seeds per paper bag based on our successful co-sowing experience. The viability of using the blended fungal powder for seed germination has been verified in our hands both *in vitro* and *in situ*. The number of seeds and the amount of powder are controllable and adjustable in each packet that can be easily preserved in the fridge for later reintroduction (Shao et al., 2017, 2018).

The interactions between the host trees for the majority of epiphytic orchids and candidate sgOMF carrying ecological specificity should also be taken into account (Martos et al., 2012; Fay et al., 2015; Rasmussen and Rasmussen, 2018). The sgOMF are susceptible to changes in moisture, pH, and organic amendments in the microhabitat provided by the host trees (McCormick et al., 2012; McCormick and Jacquemyn, 2014; Shao et al., 2018). For example, the percentage of germination of three tested *Dendrobium* species with sgOMF on the host tree *Bauhinia purpurea* was higher than that on the *Citrus maxima* and *Camellia assamica* (Shao et al., 2018).

We recognize that some orchids potentially utilize a variety of opportunistic mycorrhizal partners to induce seed germination (Waud et al., 2017). Thus, the possibility of requiring more than two strains simultaneously for *in situ* seed germination cannot be ruled out. In any case, it is necessary to experimentally identify the optimal fungus or fungal combination for seed germination leading to the re-establishment of a self-sustainable population. Following the co-sowing practice, calibrated with the aforementioned key factors, the sgOMF carrying ecological specificity in enhancing *in situ* germination are identified.

## Reintroduction With sgOMF Carrying Ecological Specificity

With the identification of sgOMF *in situ*, reintroduction can be conducted in the wild (Figure 2). The rich genetic diversity of the founder population increases the chance of successful plant colonization (Crawford and Whitney, 2010) and the functionality of the ecosystem (Prieto et al., 2015). Thus, for a given target orchid species to be reintroduced, the seeds for co-sowing with sgOMF are ideally chosen from the population with the richest genetic diversity based on assessments such as simple sequence repeats (SSR) (Zotz, 2013). A mix of several populations for one target species of origin may improve the chances of local adaptation at the recipient site. To this end, a minimum of 50–200 individuals is required to establish an effective and self-sustainable population for out-crossing species such as the majority of orchids (Reiter et al., 2016). Further, it is necessary to monitor over a long period of time to determine the sustainability of the rebuilt populations. Given the complex process involved in the re-establishment of orchid (Rasmussen et al., 2015; Shao et al., 2017), other factors including availability of pollinators (Heywood, 2015) and local rOMF (Dearnaley et al., 2012; McCormick et al., 2018) should also be taken into account for successful reintroduction (Figure 2).

## Concluding Remarks and Future Perspectives

We illustrated here the application of sgOMF selected from sOMF in the orchid reintroduction. Through performing *in situ* co-sowing with sgOMF carrying ecological specificity, coupled with other relevant measures as discussed, it is feasible to achieve an effective and self-sustained population establishment of endangered orchids in the wild. This approach rectifies the shortcomings of the current methods based on rOMF in the genus *Dendrobium* and should be now tested in more species and regions. The reintroduction of epiphytic orchids is still in its infancy, and many factors in the described protocol can be optimized further or adapted to other species. We call for more applications and updating of our method as we deeply believe that this integrated protocol provides a valuable basis for the reintroduction and protection of threatened orchid species worldwide. This could contribute to the global efforts in orchid reintroduction and realization of *in situ* restorations of threatened orchid populations in the long run.

## Author Contributions

D-KZ, S-CS, and Y-LR conceived the project. LW, YL, D-KZ, S-CS, M-AS, and Y-LR analyzed the selected references. D-KZ, S-CS, M-AS, and Y-LR wrote the manuscript with inputs from all authors. All authors read and approved the final manuscript.

## Conflict of Interest

The authors declare that the research was conducted in the absence of any commercial or financial relationships that could be construed as a potential conflict of interest.

## Abbreviations

OMF: Orchid mycorrhizal fungi
sgOMF: Seed germination-promoting orchid mycorrhizal fungi
rOMF: Root-originated OMF
sOMF: Seed-originated OMF
SSR: Simple sequence repeats.
